# The effect of octahedral distortions on the electronic properties and magnetic interactions in O3 NaTMO_2_ compounds (TM = Ti–Ni & Zr–Pd)

**DOI:** 10.1039/c8ra00576a

**Published:** 2018-04-13

**Authors:** M. Hussein N. Assadi, Yasuteru Shigeta

**Affiliations:** Center for Computational Sciences, University of Tsukuba Tennodai 1-1-1 Tsukuba Ibaraki 305-8577 Japan h.assadi.2008@ieee.org

## Abstract

The interplay between the coordination environment and magnetic properties in O3 layered sodium transition metal oxides (NaTMO_2_) is a fascinating and complex problem. Through detailed and comprehensive density functional investigations on O3 NaTMO_2_ compounds, we demonstrate that the TM ions in O3 NaMnO_2_, NaFeO_2_ and NaCoO_2_ adopt a high spin state. Structurally, NaMnO_2_ and NaPdO_2_ undergo Jahn–Teller distortions while NaNbO_2_ undergoes puckering distortion. Furthermore, in addition to Jahn–Teller distortion, NaPdO_2_ exhibits charge disproportionation as it contains Pd^2+^, Pd^3+^ and Pd^4+^ species. These distortions stabilize the inter-plane ferromagnetism. Additionally, the inter-plane ferromagnetic coupling is stabilized by kinetic p–d exchange mechanism in undistorted NaCoO_2_, NaNiO_2_ and NaTcO_2_. The intra-plane coupling in this family of compounds on the other hand was found to be generally weak. Only NaMnO_2_, NaNiO_2_ and NaTcO_2_ are predicted to show bulk ferromagnetism with estimated Curie temperatures below ∼50 K.

## Abstract 2

Although O3 sodium transition metal oxides (NaTMO_2_) share many similarities, they still differ from one another by some fine structural details.

## Graphical abstract

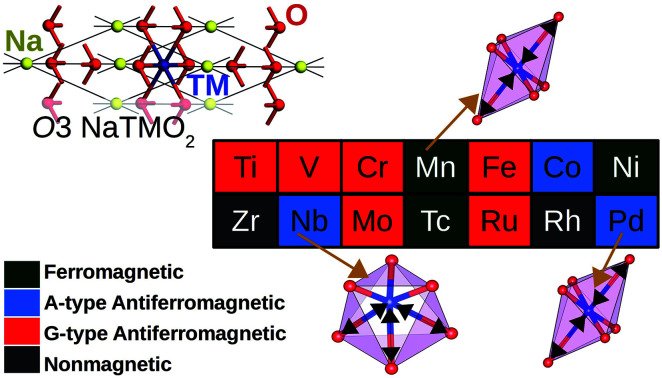

## 1. Introduction

Layered hexagonal compounds with formula NaTMO_2_ in which TM is a transition metal often exhibit interesting magnetic,^1–3^ thermoelectric,^4^ and electrochemical properties.^5^ One obstacle in studying these compounds is the rich variety of their polymorphs each with distinct symmetry and local coordination for the transition metal ions which complicates finding general property trends for this family of materials. One such area of research that lacks a concise overview is the magnetic properties of the NaTMO_2_ compounds. For instance, reports of conflicting experimental observations of the magnetic properties for the same compound is not unheard of.^6,7^ Such contradictions oftentimes stem from coarse structural characterization, restricted by instruments' range and resolution, which falls short in capturing the delicate structural details that dictate the magnetic ground state in these compounds.^8,9^ This study therefore presents a detailed and focused density functional insight into one important family of such layered materials, namely O3 NaTMO_2_ compounds in which TM is a fourth or fifth row transition metal element.

**Fig. 1:**
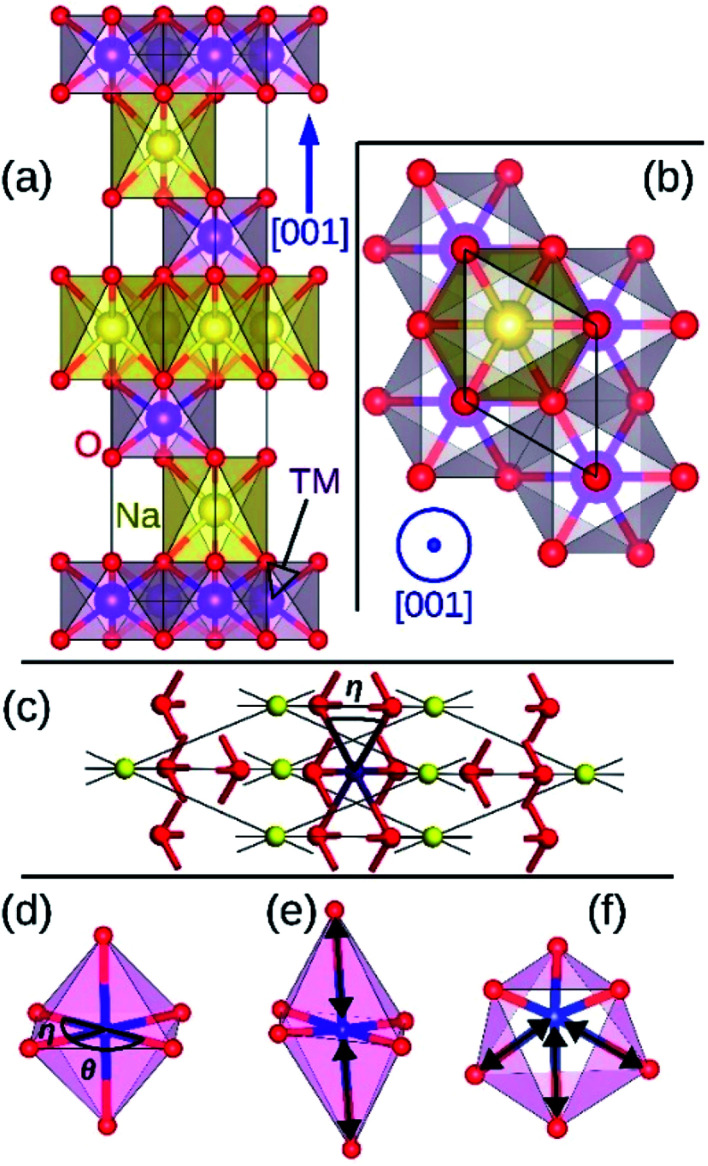
The side view (a) and the top view (b) of O3 NaTMO_2_ compounds in hexagonal representation. The same lattice structure in rhombohedral representation is shown in (c). O, TM and Na ions occupy 6c, 3b and 3a Wyckoff positions respectively. The rhombohedral, elongated Jahn–Teller and puckering distortions are presented in (d), (e) and (f) respectively. Elongated bonds are marked with double arrows.

We start our investigations with compounds of *R*3̄*m* symmetry which is a common symmetry group among layered compounds.^10^ As shown in Fig. 1(a) and (b), the hexagonal representation of this structure consists of three alternating TMO_2_ and Na layers. The notion “O3” indicates that oxygen ions are stacked in ABCABC order and Na ions occupy the octahedral site with respect to the surrounding O ions. The primitive cell of the *R*3̄*m* O3 structure, presented in Fig. 1(c), has rhombohedral symmetry and the TM ion is located in the center of the primitive cell coordinated by six oxygen ions. The O–TM–O angles, marked *η* in Fig. 1(c), depend on the lattice parameters of the rhombohedral primitive cell (*a* and *α*) and the fractional coordinates of oxygen. If this angle is not exactly 90°, then it follows that the O–TM–O angles alternate between values smaller and larger than 90° resulting in a rhombohedral distortion to the TMO_6_ octahedra. These angles are marked *η* and *θ* in Fig. 1(d). This distortion decreases the octahedral symmetry and splits the energy levels of the t_2g_ orbitals of the TM ions into a single a_1g_ orbital and doubly degenerate 
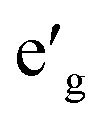
 orbitals. The sequence of stabilization of the a_1g_ and 
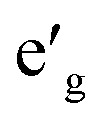
 orbitals is not however trivial.^11^ In addition to the rhombohedral distortion which is inherent to the *R*3̄*m* symmetry, NaTMO_2_ compounds may also experience additional distortions that further reduce the overall symmetry and influence the electronic structure. We will thoroughly examine all such distortions and determine how they influence the electronic and magnetic properties of O3 NaTMO_2_ compounds.

## 2. Computational and system settings

Spin polarized density functional theory (DFT) calculations were carried out within augmented plane-wave potential^12^ formalism as implemented in VASP code.^13,14^ Brillouin zone was sampled using a mesh generated by Monkhorst–Pack scheme with spacing of ∼0.02 Å^−1^ among *k* points while the energy cut-off was set to 550 eV. The threshold for energy convergence was set to 10^−7^ eV per atom. Orbital population and bonding characteristics were examined using LOBSTER code.^15^

The exchange-correlation functional was approximated by the Perdew–Burke–Ernzerhof method.^16,17^ To improve the electronic description of the compounds in term of localizing the d shell electrons of the transition metal elements, an orbital dependent Hubbard term^18^ was applied to the d orbitals. The value of *U*_eff_(*U* − *J*) was set to 5 eV for 3d TM elements and 2 eV for 4d TM elements. Weaker localization effects of the 4d electrons justifies the smaller *U*_eff_ value for the 4d elements. Among the different elements of the 3d and the 4d rows, a small variation in *U*_eff_ is naturally expected. However, the choice of constant *U*_eff_ for each row allows a more straightforward comparison.^19^ This procedure is further justified by the fact that the localization effects in NaVO_2_ are not affected by slight variation of *U*_eff_.^11^ Furthermore, as shown in Table 1, the applied *U*_eff_ values reproduce the lattice constants reported in earlier experiments within ∼1% deviation indicating the adequacy of the chosen values.

**Table 1:** Calculated and observed lattice parameters and structural data for NaTMO_2_ compounds in hexagonal representation

System	Calculated *a* (Å)	Calculated *c* (Å)	Experimental *a* (Å)	Experimental *c* (Å)	Ref.	TM–O (Å)	*η* (°)
NaTiO_2_	3.042	16.551	3.037	16.260	10	2.11	92.25
NaVO_2_	3.055	16.242	2.996	16.100	11	2.10	95.57
NaCrO_2_	3.052	16.146	3.030	16.000	20	2.06	95.66
NaMnO_2_	3.087^a^	16.234	—	—	—	2.01, 2.26	92.16^b^
NaFeO_2_	3.061	16.286	3.029	16.113	21	2.03	94.86
NaCoO_2_	2.908	15.776	2.891	15.612	21	1.95	96.13
NaNiO_2_	3.000	15.899	2.960	15.780	6	2.02	95.89
NaZrO_2_	3.206	17.217	—	—	—	2.24	90.63
NaNbO_2_	2.998^a^	17.817	—	—	—	2.22 (unpuckered), 2.18 (puckered), 2.23 (puckered)	91.15^b^
NaMoO_2_	3.272	16.128	—	—	—	2.19	96.90
NaTcO_2_	3.111	16.466	—	—	—	2.14	93.12
NaRuO_2_	3.121	15.968	3.124	16.037	22	2.11	96.29
NaRhO_2_	3.151	15.725	3.097	15.527	23	2.10	97.03
NaPdO_2_	3.235^a^	15.854	—	—	—	2.07 (Pd^4+^), 2.10 (Pd^3+^), 2.32 (Pd^3+^), 2.27 (Pd^2+^)	90.00^b^

a The lattice parameter *a* of the supercell has been divided by the number of hexagonal unit cells in corresponding dimensions of the supercell.

b These values correspond to the angles closest in value to 90° in distorted systems.

O3 NaTMO_2_ structure with *R*3̄*m* symmetry in hexagonal representation, as shown in Fig. 1(a) and (b), was initially used for all compounds. To find the final geometries of NaTMO_2_ compounds, the lattice parameters and all internal coordinates of the primitive cell were allowed to relax to forces smaller than 0.001 eV Å^−1^. Furthermore, the geometry optimization was repeated with 2*a* × 2*a* × 1*c*, 3*a* × 3*a* × 1*c* and 4*a* × 4*a* × 1*c* supercells with symmetry restrictions turned off, to detect any possible distortion that may lower the total energy by breaking the symmetry.

The magnetic phase stability was examined by comparing the total energies of the ferromagnetic system (*E*^t^_FM_) with those of competing antiferromagnetic phases. The energy of the ferromagnetic state was calculated by aligning the spin of the all TM ions in the hexagonal cell parallel. The total energy of the C-type antiferromagnetic state (*E*^t^_CAFM_) was calculated by aligning the spin of adjacent TM ions within the basal planes of a 2*a* × 1*a* × 1*c* supercell antiparallel. Δ*E*_CAFM_ is defined as the difference between total energies *E*^t^_CAFM_ and *E*^t^_FM_ the per TM ion:

1 Δ*E*_CAFM_ = [(*E*^t^_CAFM_/2) − *E*^t^_FM_]/*n*

Here, *n* is the total number of the TM ions in the ferromagnetic supercell which is 3 for systems without distortions but larger for distorted systems. The energy of the A-type antiferromagnetic states (*E*^t^_AAFM_) calculated by aligning the spin of TM ions in a 1*a* × 1*a* × 2*c* supercell antiparallel in alternating manner. Δ*E*_AAFM_ is defined as the difference between *E*^t^_AAFM_ and *E*^t^_FM_ the per TM ion:

2 Δ*E*_AAFM_ = [(*E*^t^_AAFM_/2) − *E*^t^_FM_]/*n*

Positive Δ*E*_CAFM_ values indicate the preference of TM ion to align ferromagnetically within a TMO_2_ plane (inter-plane) while positive *E*^t^_AAFM_ indicate the preference of ferromagnetic coupling across TMO_2_ planes (intra-plane).

## 3. Results and discussions

### 3.1. TM spin state

Based on the calculated spin populations presented in Table 2, the early 3d TM ions in NaTiO_2_, NaVO_2_ and NaCrO_2_ generally conform to the octahedral crystal field splitting t_2g_e_g_. However, as we will see later, there are finer splittings among t_2g_ states caused by symmetry considerations. Later TM ions in NaMnO_2_, NaFeO_2_ and NaCoO_2_ compounds stabilize in high spin configuration. We found that the total energy of the NaMnO_2_ compound rose by 1.306 eV/f.u. (f.u. is formula unit) when the Mn ion was set to low spin configuration (t_2g_^4^e^0^_g_). Similarly, the total energy of low spin NaFeO_2_ (t_2g_^5^e^0^_g_) rose by 0.889 eV/f.u. and the total energy of low spin NaCoO_2_ (t_2g_^6^e^0^_g_) rose by 0.157 eV/f.u. with respect to their corresponding high spin configurations. Ni ions in NaNiO_2_, nonetheless, are stabilized in low spin configuration as setting Ni to high spin configuration (t_2g_^5^e_g_^2^) raised the total energy by 0.777 eV/f.u. Our calculations for Ni is agreement with the experimental observation of low spin NaNiO_2_.^24^

**Table 2:** Nominal electronic configuration of the d shell in NaTMO_2_ compound, calculated number of unpaired d electrons (spin population) and the energy difference between ferromagnetic and antiferromagnetic states (Δ*E*) are given. The nominal electronic configuration corresponds to the hypothetical complete ionic bonding. The magnetic ground state and the conduction type of all compounds also summarized here. FM, AAFM, GAFM stand for ferromagnetic, A-type and G-type antiferromagnetic states respectively

Compound	Nominal configu-ration	Calculated unpaired d electrons	Δ*E*_CAFM_ (mEV)	Δ*E*_AAFM_ (mEV)	Magnetic ground state	Conduction
NaTiO_2_	t_2g_^1^e^0^_g_	0.897	−395.365	−0.228	GAFM	Insulator
NaVO_2_	t_2g_^2^e^0^_g_	1.879	−18.163	−3.204	GAFM	Insulator
NaCrO_2_	t_2g_^3^e^0^_g_	2.925	−4.341	−0.323	GAFM	Insulator
NaMnO_2_	t_2g_^3^e_g_^1^	3.922	51.657	0.197	FM	Insulator
NaFeO_2_	t_2g_^3^e_g_^2^	4.277	−4.738	−1.894	GAFM	Insulator
NaCoO_2_	t_2g_^4^e_g_^2^	3.149	152.238	−2.410	AAFM	Half metallic
NaNiO_2_	t_2g_^6^e_g_^1^	1.378	24.564	0.826	FM	Half metallic
NaZrO_2_	t_2g_^1^e^0^_g_	0.000	—	—	Nonmagnetic	Metallic
NaNbO_2_	t_2g_^2^e^0^_g_	1.110, 0.350	14.468	−1.240	AAFM	Metallic
NaMoO_2_	t_2g_^3^e^0^_g_	2.567	−117.673	−2.272	GAFM	Insulator
NaTcO_2_	t_2g_^4^e^0^_g_	1.721	65.860	2.565	FM	Half metallic
NaRuO_2_	t_2g_^5^e^0^_g_	0.858	−13.462	−5.030	GAFM	Insulator
NaRhO_2_	t_2g_^6^e^0^_g_	0.000	—	—	Nonmagnetic	Insulator
NaPdO_2_	t_2g_^6^e_g_^2^	1.339	11.315	−2.750	AAFM	Insulator
	t_2g_^6^e_g_^1^	0.621				
	t_2g_^6^e^0^_g_	0.015				

Unlike their 3d counterparts, early 4d TM ions in NaZrO_2_ and NaNbO_2_, deviate from t_2g_e_g_ splitting as Zr bears no magnetic moment and Nb adopts two distinct magnetic moments both significantly smaller than the anticipated t_2g_^2^e^0^_g_. NaZrO_2_ in which Zr set to t_2g_^1^e^0^_g_ was 0.317 eV/f.u. higher in energy than non-magnetic NaZrO_2_ while NaNbO_2_ with Nb fixed to t_2g_^2^e^0^_g_ configuration was 0.649 eV/f.u. higher than the presented ground state. Moreover, contrarily to the 3d TM ions, the later 4d TM ions in NaTMO_2_ stabilized in low spin configuration. The total energy of NaTcO_2_ rose by 1.086 eV/f.u. when Tc was set to high spin configuration (t_2g_^2^e_g_^2^). Similarly, the high spin NaRuO_2_ (t_2g_^3^e_g_^2^) and NaRhO_2_ (t_2g_^4^e_g_^2^) were higher in energy than their low spin counterpart by 1.937 eV/f.u. and 4.642 eV/f.u. respectively.

### 3.2. Electronic structures

**Fig. 2:**
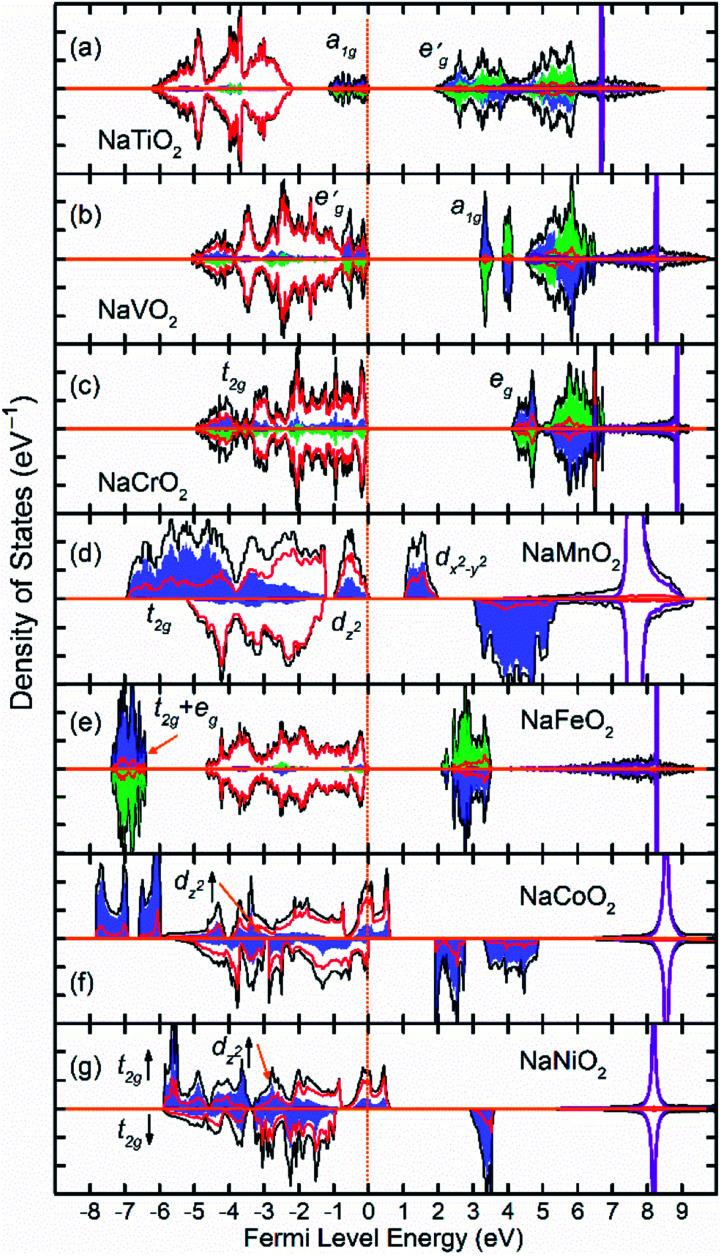
The density of states (DOS) of NaTMO_2_ compounds in which TM is forth row transition metal element. The black, red and purple lines correspond to total, O 2p and Na states respectively. The blue and green shaded areas correspond to TM 3d elements with net spin-up and spin-down electronic population respectively. Compounds with only blue shaded areas are either non-magnetic or those with inter-plane ferromagnetic coupling.

Fig. 2 shows the total and partial density of states (DOS) of 3d TM containing NaTMO_2_ compounds. In the NaTiO_2_, rhombohedral distortion in the TiO_6_ octahedra splits the t_2g_ orbitals of the spin-up channel into lower single fold a_1g_ orbital which is occupied by Ti^3+^’s lone 3d electron and higher empty 
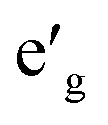
 orbitals. Furthermore, a_1g_ orbital is detached from the lower O 2p states and creates a pseudo-gap within the valence band. Consequently, the complete separation of Ti 3d states from O 2p states implies that Ti–O bond is highly ionic. In NaVO_2_, the rhombohedral splitting is still dominant. However, contrary to the Ti case, the occupied 
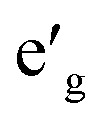
 orbitals have lower energy than the empty a_1g_ orbital. Moreover, since the gap between 
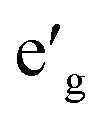
 states and O 2p states is now closed, there is a greater hybridization between 
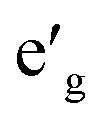
 and O 2p states which reduces the ionicity of the V–O bond compared to that of Ti–O bond. In NaCrO_2_, through the merging of the 
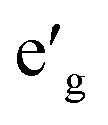
 and a_1g_ states in the spin-up channel, the band structure resembles conventional octahedral splitting where the spin-up t_2g_ states in the valence band are all occupied while the empty e_g_ states constitute the bottom of the conduction band. The DOS of NaMnO_2_ corresponds to the elongated Jahn–Teller distortion. The lower region of the valence band (∼−7 eV < *E* < ∼−4 eV) is occupied by d_*xz*_ and d_*yz*_ states while the middle part (∼−4 eV < *E* < ∼−1.2) is occupied by d_*xy*_ states. The top of valence band is nevertheless occupied by d_*z*^2^_ states. As inferred from the DOS, the proximity of d_*xy*_ and d_*z*^2^_ favors the high spin configuration for the Mn ions.

The DOS of the half-filled Fe 3d shell (t^3^_2g_e^2^_g_) in NaFeO_2_ exhibits a different arrangement when compared to earlier compounds. Here, due to strong electron–electron repulsion between the half-filled Fe 3d^5^ states and O 2p states, all of the occupied Fe 3d states are shifted downwards below O 2p states. The proximity of the t^3^_2g_ and e^2^_g_ states in the spin-up channel to one another favors the high spin configuration for Fe ions as the spin-down t_2g_ states are ∼11 eV higher in energy than spin-up e_g_ states. In NaCoO_2_, the t_2g_ states of the spin-up channel, although mainly concentrate at the bottom of the valence band, still stretch over the entire valence band width and strongly hybridize with O 2p states. Furthermore, the tale of the spin-up t_2g_ states crosses the Fermi level creating half metallic conduction. Similarly, in NaNiO_2_, the spin-up t_2g_ states stretch over the valence band and cross the Fermi level while the spin-down t_2g_ states and d_*z*^2^_ states remain confined within the middle of the valence band without crossing the Fermi level.

**Fig. 3:**
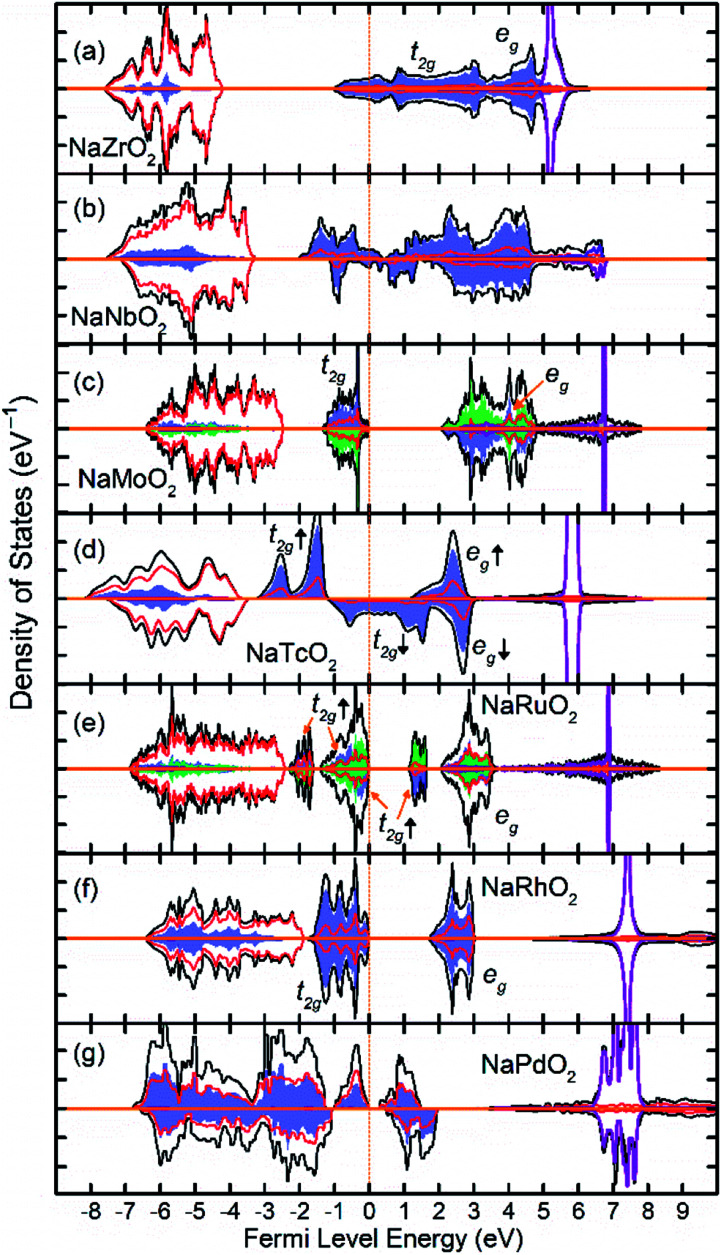
The density of states (DOS) of NaTMO_2_ compounds in which TM is fifth row transition metal element. The black, red and purple lines correspond to total, O 2p and Na states respectively. The blue and green shaded areas correspond to TM 4d elements with net spin-up and spin-down electronic population respectively. Compounds with both blue and green shaded areas are G-type antiferromagnetic.

Fig. 3 show the total and partial DOS in 4d TM containing compounds. NaZrO_2_ exhibits strong metallic character as its Fermi level intercepts the Zr 4d states in the conduction band. Metallicity in NaZrO_2_ is facilitated by a metallic Zr–Zr bond which is caused by extraordinarily large Zr^3+^ ionic radius of ∼0.89 Å (ref. 25) and the Zr–Zr distance of 3.21 Å which is comparable to that in Zr metal. The metallic character of NaZrO_2_ explains the lack of magnetic moment as there is no significant hybridization between Zr with O. NaNbO_2_ also exhibits metallic conduction as the Fermi level crosses through the 4d states in the conduction band. However, as we will discuss later, due to puckering distortion, there are two distinct Nb species in this compound each with different levels of metallicity. The band structure of the NaMoO_2_ shows a conventional octahedral distortion where the half-filed t_2g_ states constitute the top of the valence band while the empty e_g_ states are separated by ∼1 eV above the Fermi level. In NaTcO_2_, NaRuO_2_ and NaRhO_2_ compounds the spin-down channel of the t_2g_ states is progressively filled as expected for the TM ions in low spin configuration. As will be discussed in detail in the next section, Pd in NaPdO_2_ undergoes charge disproportionation and form Pd^2+^, Pd^3+^, Pd^4+^ species. The t_2g_ states of all Pd species occupy the lower part of the conduction band while the filled e_g_ occupy the top of the valence band.

### 3.3. Octahedral distortions

**Fig. 4:**
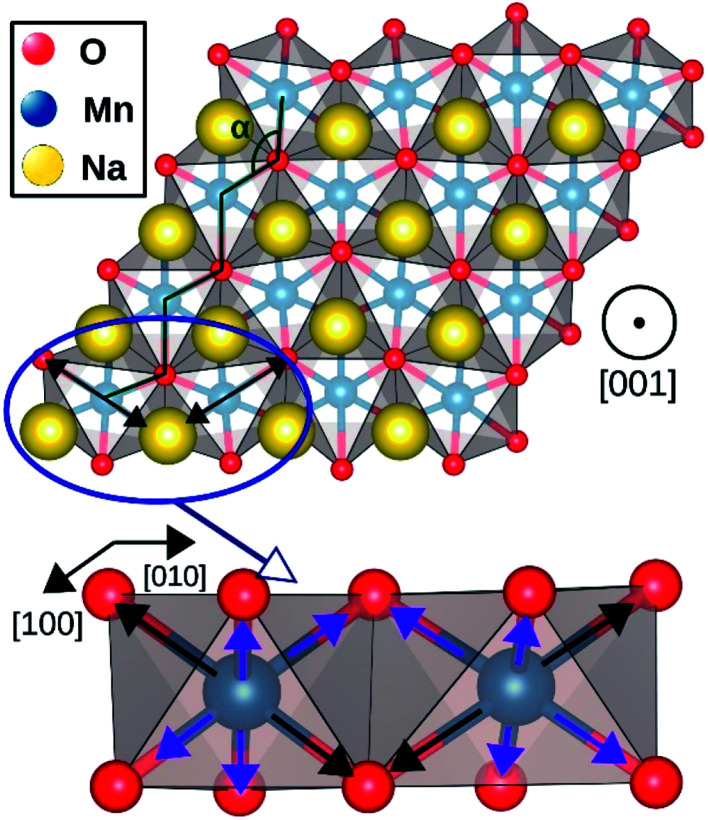
Jahn–Teller distortion in NaMnO_2_ system. The elongated bonds are marked with black arrows while the shorter bonds are marked with purple arrows.

The geometry optimization conducted with larger supercells revealed that NaMnO_2_, NaNbO_2_ and NaPdO_2_ compounds, each to a different extent, exhibits additional distortions in their TMO_6_ octahedra. In NaMnO_2_, as indicated by purple arrows in Fig. 4, two out of six Mn–O bonds in all MnO_6_ octahedra are elongated causing a deviation from perfect *R*3̄*m* symmetry. This distortion is similar to the Jahn–Teller distortion depicted in Fig. 1(e). The long Mn–O bond is 2.26 Å while the short Mn–O bonds is 2.01 Å implying a 12.4% elongation. We found that perfectly *R*3̄*m* symmetric NaMnO_2_ primitive cell with no elongation had a total energy 0.763 eV/f.u. higher than the distorted compound indicating that this distortion leads to significant stabilization.

**Fig. 5:**
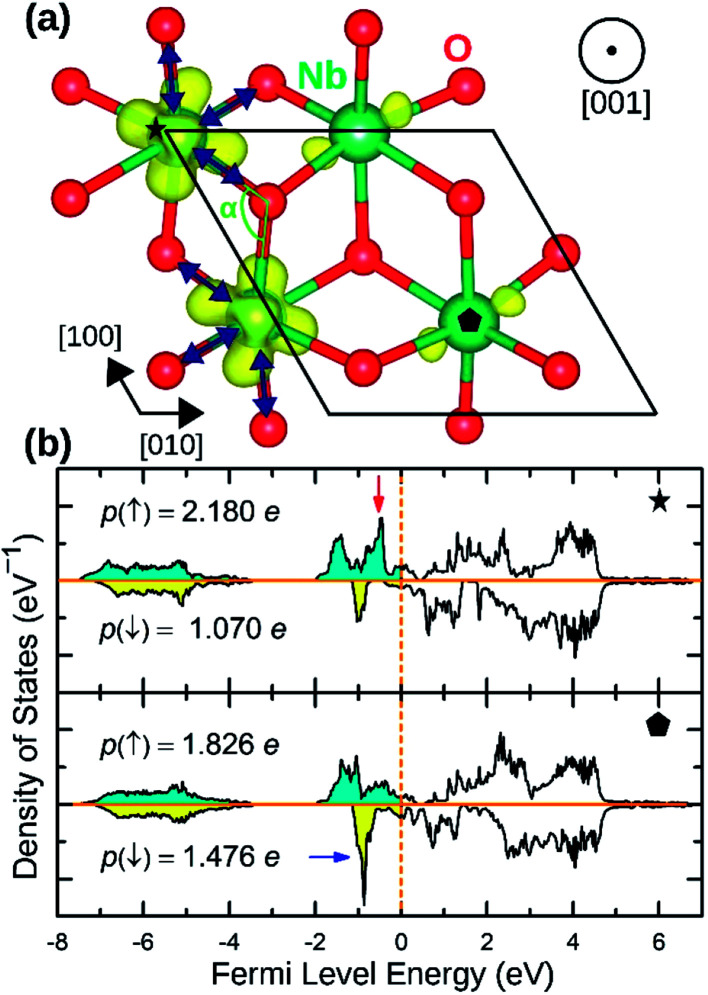
(a) The spin density isosurface of NaNbO_2_ drown at 0.025 e Å^−2^. Half of the NbO_6_ octahedra undergo puckering distortion. The compressed bonds are marked with blue arrows. (b) The site-projected Nb 4d states for high magnetization (top panel) and low magnetization (bottom panel) Nb species.

NaNbO_2_ showed puckering distortion [Fig. 1(f)] in half of its NbO_6_ octahedra. As marked by blue arrows in Fig. 5(a), NbO_6_ octahedra on every second row in [100] direction are alternately puckered to the left and the right along [010] direction while the octahedra on the adjacent row only had rhombohedral distortion. In the puckered NbO_6_ octahedra, the short Nb–O bond was 2.18 Å while the long Nb–O bond was 2.23 Å indicating a 2.2% puckering distortion in bond lengths. The bond length in unpuckered NbO_6_ octahedra had a median value of 2.22 Å. The puckering altered the electronic structure of the NaNbO_2_ compound as ions in the puckered and unpuckered octahedra had distinct spin populations of 1.051e and 0.350e respectively. According to Fig. 5(b), Nb ions in the puckered octahedra has a significantly larger spin-up population (marked with red arrow) and smaller spin-down population (marked with blue arrow) compared to the Nb ions in unpuckered octahedra. To examine the stability induced by this distortion, we once set all Nb ions to low magnetization equal to that in the unpuckered octahedra and once again to high magnetization equal to that in the puckered octahedra and recalculated the total energy. The earlier setting raised the total energy of NaNbO_2_ compound by 0.230 eV/f.u. while the latter setting raised the total energy by 0.649 eV/f.u. demonstrating the stabilizing effect of puckering distortion. Given that Nb's total electronic population does not significantly depend on the puckering of NbO_6_ octahedra, we infer that this relatively minor distortion does not cause charge disproportionation but rather only alters the magnetization of Nb ions.

**Fig. 6:**
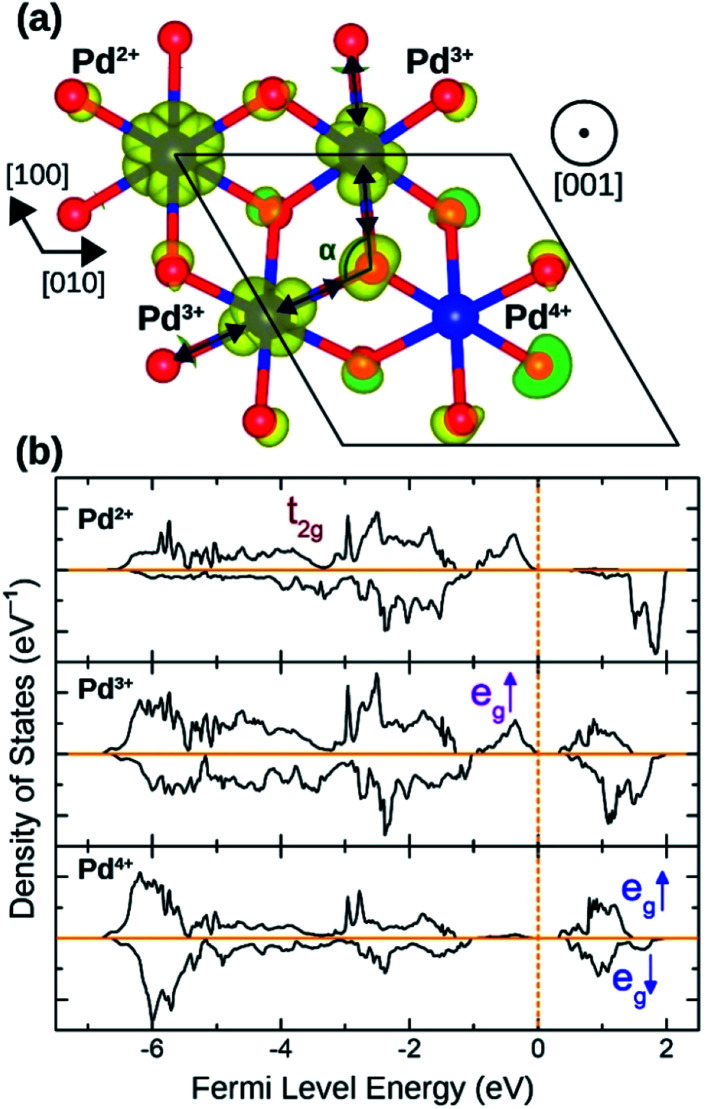
(a) The spin density isosurface in NaPdO_2_ drown at 0.025 e Å^−2^ demonstrating charge disproportionation. (b) The site-projected 4d states for Pd^2+^, Pd^3+^ and Pd^4+^ species.

The distortion in PdO_6_ octahedra in NbPdO_2_ were accompanied with charge disproportionation among Pd ions. As shown in Fig. 6(a), within a Pd containing plane perpendicular to the [001] direction in the 2*a* × 2*a* × 1*c* supercell, two Pd^3+^ ions transform into a pair of Pd^4+^ and Pd^2+^ along [110] direction while the other two Pd^3+^ ions along [11̄0] direction remain unchanged. The Pd^2+^O_6_ and Pd^4+^O_6_ octahedra had perfect octahedral symmetry however each with a different Pd–O bond length. Pd^2+^–O bond was 2.27 Å long while Pd^4+^–O bond was 2.07 Å long. The two Pd^3+^O_6_ octahedra, on the other hand, showed elongated Jahn–Teller distortion with long bonds of 2.32 Å and short bonds of 2.09 Å accounting for an elongation of 11.0%. The stability of this distortion was verified by the fact that the NaPdO_2_ in which all Pd ions were fixed to +3 oxidation state had a total energy 0.381 eV/f.u. higher than the presented ground state. Furthermore, this distortion pattern and the accompanying charge disproportionation prevailed in larger 4*a* × 4*a* × 1*c* supercell and persisted under different *U*_eff_ values.

Fig. 6(b) shows how Pd e_g_ states are occupied as charge disproportionation occurs. For Pd^2+^, e_g_'s spin-up states are all occupied while the empty spin-down states are entirely located ∼1 eV above the Fermi level. For Pd^3+^, the spin-up peak decreases (marked with a red arrow) while an empty spin-up e_g_ peak appears above the Fermi level. Finally, for Pd^4+^, all e_g_ states are now located above the Fermi level.

### 3.4. Magnetic coupling

The magnetic ground state of a compound can be predicted by comparing the total energies corresponding to different spin alignments among the TM ions that is Δ*E*_CAFM_ and Δ*E*_AAFM_.^26^ If different spin alignments result in the same energy, the compound is paramagnetic.^27^ Furthermore, Δ*E*_CAFM_ and Δ*E*_AAFM_ are functions of the magnetic exchange integrals (*J*) which determine the Curie and Néel temperatures (*T*_C_ and *T*_N_) in compounds with long range magnetic ordering.^28^ According to the mean field approximation these critical temperatures depends linearly on the *J*.^29^ For instance, room temperature ferromagnetism requires positive Δ*E*_CAFM_ and Δ*E*_AAFM_ values of ∼12 meV.^30^

As presented in Table 2, NaMnO_2_, NaCoO_2_, NaNiO_2_, NaNbO_2_ NaTcO_2_ and NaPdO_2_ have positive Δ*E*_CAFM_ values indicating inter-plane ferromagnetism which is defined as the ferromagnetic coupling among TM ions within the basal TMO_2_ planes. This ferromagnetic coupling can be attributed to one of two distinct mechanisms: the kinetic p–d exchange interaction and the superexchange interaction. The density of states in Fig. 2(f)–(g) and Fig. 3(d) reveals a special p–d hybridization in NaCoO_2_, NaNiO_2_ and NaTcO_2_ compounds. Because of this hybridization, the spin majority p states are shifted to higher energies, while the spin minority p states are shifted to lower energies. This hybridization scheme therefore creates spin polarized p states which mediate the ferromagnetic coupling.^29^ In NaMnO_2_, NaNbO_2_ and NaPdO_2_, on the other hand, positive Δ*E*_CAFM_ values are caused by ferromagnetic superexchange. The prerequisite for ferromagnetic superexchange is a ∼90° TM–O–TM angle which stabilizes the ferromagnetic coupling through π TM–O bonds in TM–O–TM trimer.^31^ The octahedral distortions in these compounds orient the TM–O–TM angles in these compounds to ∼90°. Under perfect *R*3̄*m* symmetry, as shown in Fig. 1(c), the TM–O–TM angle is determined by O's fractional coordinates and alternates between the supplementary angles *η* and *θ* [defined in Fig. 1(c) and (d)] preventing the stabilization of the ferromagnetic phase.^9,11,32,33^ If a distortion, however, breaks the symmetry and brings the TM–O–TM angle closer to 90°, ferromagnetic superexchange can prevail. One should note that, as indicated in Table 1, the octahedral distortions in these compounds basically bring the O–TM–O angle closer to 90°. However, since these compounds do not have any octahedral tilting, the TM–O–TM angle, at least in certain planes, also approaches 90° due to the similar distortion in neighbouring TMO_6_ octahedra. Those TM–O–TM angles assisting the ferromagnetic superexchange are marked *α* in Fig. 4–6. *α* is 91.67° in NaMnO_2_, 89.86° in NaNbO_2_ and 88.48° in NaPdO_2_. Contrary to our results, earlier DFT calculation of the NaMnO_2_ compound using a small supercell restricted to *C*2/*m* symmetry, predicted weak frustrated antiferromagnetic ground state.^34^ This discrepancy shows the importance of taking into account the octahedral distortions that stabilizes the ferromagnetic ground state. Inferred from Δ*E*_CAFM_ values, the kinetic p–d exchange interaction seems to be generally stronger than the ferromagnetic superexchange interaction.

The magnetic coupling across the TMO_2_ planes or intra-plane coupling, in principle, can be mediated a by second nearest neighbour superexchange interaction through TM–O–Na–O–TM chain *via* by O's p orbitals and Na sp^2^ hybrid orbitals.^35^ Because of anisotropy in the *R*3̄*m* crystal structure which prevents the hybridization of TM d states with the p states of adjacent TMO_2_ layers, p–d kinetic exchange is not expected to result in significant intra-plane coupling. Among all compounds only NaMnO_2_, NaNiO_2_ and NaTcO_2_ had small positive Δ*E*_AAFM_ values indicating weak ferromagnetic intra-plane coupling resulting in *T*_C_ values lower than ∼50 K. This prediction, particularly for the NaNiO_2_ compound, is agreement with the earlier observation that measured a *T*_C_ of ∼20 K.^7^ In the case of NaNbO_2_, NaPdO_2_ and NaCoO_2_ compounds, the negative Δ*E*_AAFM_ values along with positive Δ*E*_CAFM_ values predict A-type antiferromagnetic ground state. For the rest of compounds for which both Δ*E*_AAFM_ and Δ*E*_CAFM_ are negative, G-type antiferromagnetic ground state prevails. Such antiferromagnetism has been observed in NaCrO_2_ with *T*_N_ = 40–50 K,^36,37^ NaVO_2_ (ref. 11 and 38) and NaTiO_2_ (ref. 39).

Last, note that relativistic spin–orbit interaction can play a significant role in determining the structural and magnetic properties of isolated TM octahedral complexes.^40–43^ However, in the context of bulk NaTMO_2_ compounds that have been studied here, the role of spin–orbit interaction on the calculated Δ*E*_CAFM_ and Δ*E*_AAFM_ values is negligibly small. Spin–orbit interaction constant is proportional to the mass of the interacting ions and can be significant in 5d TM oxides such as iridates.^44^ However, experimental studies have shown that the magnitude of the spin–orbit interaction in 3d and 4d TM oxides such as cobaltates^45^ and rhoates^46^ is generally small. To verify this notion, we recalculated the Δ*E*_CAFM_ and Δ*E*_AAFM_ for NaPdO_2_ with the inclusion of the spin–orbit calculation and obtained Δ*E*_CAFM_ = 11.492 meV and Δ*E*_AAFM_ = −2.841 meV. These values differ only by ∼0.1 meV from the values of Table 2 which have been obtained without including spin–orbit interaction. The role of spin–orbit interaction is expected to be even smaller for the rest of the compounds, especially for 3d NaTMO_2_, as their molecular mass is considerably smaller than that of NaPdO_2_.

## 4. Conclusions

We demonstrated that the rhombohedral distortion inherent to the 3*R̄m* symmetry stabilizes G-type antiferromagnetism in NaTiO_2_, NaVO_2_, NaCrO_2_, NaFeO_2_, NaMoO_2_ and NaRuO_2_ compounds. Inter-plane ferromagnetism however can be stabilized if the 3*R̄m* symmetry breaks due to additional octahedral distortions which is the case for NaMnO_2_, NaNbO_2_ and NbPdO_2_. Here, because of favorable orbital orientation, the superexchange interaction stabilizes ferromagnetism instead of antiferromagnetism among the TM ions of the same TMO_2_ plane. Additionally, strong p–d hybridization, as in NaCoO_2_, NaNiO_2_ and NaTcO_2_ can also result in inter-plane ferromagnetism. In this case, due to its strength, the kinetic p–d exchange mechanism overcomes the underlying inter-plane antiferromagnetism. The intra-plane ferromagnetic coupling is mediated by a weak second neighbor coupling which prevails only in NaMnO_2_, NaTcO_2_ and NaNiO_2_ giving rise to bulk ferromagnetism with low *T*_C_.

The weak intra-plane coupling appears to be a general feature of O3 compounds. This is in contrast to the P2 structures in which the magnitudes of inter-plane and intra-plane coupling are of the same order.^35^ This is probably because this interaction strongly depends on Na's coordination environment. This line of enquiry warrants further research.

## Conflicts of interest

The authors declare no competing financial interest.

## Supplementary Material

## References

[cit1] Mostovoy M. V., Khomskii D. I. (2002). Phys. Rev. Lett..

[cit2] Velikokhatnyi O. I., Chang C.-C., Kumta P. N. (2003). J. Electrochem. Soc..

[cit3] Viciu L., Bos J. W. G., Zandbergen H. W., Huang Q., Foo M. L., Ishiwata S., Ramirez A. P., Lee M., Ong N. P., Cava R. J. (2006). Phys. Rev. B: Condens. Matter Mater. Phys..

[cit4] Walia S., Balendhran S., Nili H., Zhuiykov S., Rosengarten G., Wang Q. H., Bhaskaran M., Sriram S., Strano M. S., Kalantar-zadeh K. (2013). Prog. Mater. Sci..

[cit5] Slater M. D., Kim D., Lee E., Johnson C. S. (2013). Adv. Funct. Mater..

[cit6] Chappel E., Nunez-Regueiro M. D., Dupont F., Chouteau G., Darie C., Sulpice A. (2000). Eur. Phys. J. B.

[cit7] Brion S. d., Bonda M., Darie C., Bordet P., Sheikin I. (2010). J. Phys.: Condens. Matter.

[cit8] Kaplan T. A., Menyuk N. (2007). Philos. Mag..

[cit9] Terada N., Ikedo Y., Sato H., Khalyavin D. D., Manuel P., Miyake A., Matsuo A., Tokunaga M., Kindo K. (2017). Phys. Rev. B: Condens. Matter Mater. Phys..

[cit10] Wu D., Li X., Xu B., Twu N., Liu L., Ceder G. (2015). Energy Environ. Sci..

[cit11] Jia T., Zhang G., Zeng Z., Lin H. Q. (2009). Phys. Rev. B: Condens. Matter Mater. Phys..

[cit12] Kresse G., Joubert D. (1999). Phys. Rev. B: Condens. Matter Mater. Phys..

[cit13] Kresse G., Furthmüller J. (1996). Phys. Rev. B: Condens. Matter Mater. Phys..

[cit14] Kresse G., Furthmüller J. (1996). Comput. Mater. Sci..

[cit15] Maintz S., Deringer V. L., Tchougréeff A. L., Dronskowski R. (2016). J. Comput. Chem..

[cit16] Perdew J. P., Burke K., Ernzerhof M. (1996). Phys. Rev. Lett..

[cit17] Perdew J. P., Burke K., Ernzerhof M. (1997). Phys. Rev. Lett..

[cit18] Dudarev S., Botton G., Savrasov S., Humphreys C., Sutton A. (1998). Phys. Rev. B: Condens. Matter Mater. Phys..

[cit19] Gopal P., Spaldin N. A. (2006). Phys. Rev. B: Condens. Matter Mater. Phys..

[cit20] Zhou Y.-N., Ding J.-J., Nam K.-W., Yu X., Bak S.-M., Hu E., Liu J., Bai J., Li H., Fu Z.-W., Yang X.-Q. (2013). J. Mater. Chem. A.

[cit21] Kubota K., Asari T., Yoshida H., Yaabuuchi N., Shiiba H., Nakayama M., Komaba S. (2016). Adv. Funct. Mater..

[cit22] Mogare K. M., Friese K., Klein W., Jansen M. (2004). Z. Anorg. Allg. Chem..

[cit23] Hobbie K., Hoppe R. (1988). Z. Anorg. Allg. Chem..

[cit24] Chappel E., Núñez-Regueiro M. D., Chouteau G., Isnard O., Darie C. (2000). Eur. Phys. J. B.

[cit25] Jeon S., Ryu J., Shin H.-G., Lee J., Lee H. (2017). Mater. Charact..

[cit26] Sato K., Bergqvist L., Kudrnovský J., Dederichs P. H., Eriksson O., Turek I., Sanyal B., Bouzerar G., Katayama-Yoshida H., Dinh V. A., Fukushima T., Kizaki H., Zeller R. (2010). Rev. Mod. Phys..

[cit27] Assadi M. H. N., Katayama-Yoshida H. (2015). Funct. Mater. Lett..

[cit28] Lee J. , PhD thesis, The University of Texas at Austin, 2010

[cit29] Belhadji B., Bergqvist L., Zeller R., Dederichs P. H., Sato K., Katayama-Yoshida H. (2007). J. Phys.: Condens. Matter.

[cit30] Anderson P. W. , in Solid State Physics, ed. F. Seitz and D. Turnbull, Academic Press, 1963, vol. 14, pp. 99–214

[cit31] Coey J. M. D. , Venkatesan M. and Xu H., in Functional Metal Oxides, Wiley-VCH Verlag GmbH & Co. KGaA, 2013, pp. 1–49, 10.1002/9783527654864.ch1

[cit32] Takada K., Sakurai H., Takayama-Muromachi E., Izumi F., Dilanian R. A., Sasaki T. (2003). Nature.

[cit33] Bayrakci S. P., Mirebeau I., Bourges P., Sidis Y., Enderle M., Mesot J., Chen D. P., Lin C. T., Keimer B. (2005). Phys. Rev. Lett..

[cit34] Zhang G. R., Zou L. J., Zeng Z., Lin H. Q. (2009). J. Appl. Phys..

[cit35] Johannes M. D., Mazin I. I., Singh D. J. (2005). Phys. Rev. B: Condens. Matter Mater. Phys..

[cit36] Hsieh D., Qian D., Berger R. F., Cava R. J., Lynn J. W., Huang Q., Hasan M. Z. (2008). Phys. B.

[cit37] Olariu A., Mendels P., Bert F., Ueland B. G., Schiffer P., Berger R. F., Cava R. J. (2006). Phys. Rev. Lett..

[cit38] McQueen T. M., Stephens P. W., Huang Q., Klimczuk T., Ronning F., Cava R. J. (2008). Phys. Rev. Lett..

[cit39] Yamada I., Ubukoshi K., Hirakawa K. (1985). J. Phys. Soc. Jpn..

[cit40] David J., Restrepo A. (2007). Phys. Rev. A.

[cit41] David J., Fuentealba P., Restrepo A. (2008). Chem. Phys. Lett..

[cit42] Pérez-Villa A., David J., Fuentealba P., Restrepo A. (2011). Chem. Phys. Lett..

[cit43] David J., Guerra D., Restrepo A. (2011). Inorg. Chem..

[cit44] Singh Y., Manni S., Reuther J., Berlijn T., Thomale R., Ku W., Trebst S., Gegenwart P. (2012). Phys. Rev. Lett..

[cit45] Yanase Y., Mochizuki M., Ogata M. (2005). J. Phys. Soc. Jpn..

[cit46] Mazin I. I., Manni S., Foyevtsova K., Jeschke H. O., Gegenwart P., Valentí R. (2013). Phys. Rev. B: Condens. Matter Mater. Phys..

